# RISEinFAMILY project: the integration of families at neonatal intensive care units (NICUs) to empower them as primary caregivers: study protocol for a stepped wedge cluster controlled trial

**DOI:** 10.1186/s13063-024-08043-7

**Published:** 2024-04-10

**Authors:** M. T. Alferink, B. Moreno-Sanz, M. Cabrera-Lafuente, E. Ergenekon, T. R. de Haan, A. A. M. W. van Kempen, J. Lakhwani, H. Rabe, G. C. Zaharie, A. Pellicer, on behalf of the RISEinFAMILY Consortium (European Union, MSCA-RISE-H2020)

**Affiliations:** 1 Division of Pediatrics/Neonatology, OLVG, Location East and West, Amsterdam, The Netherlands; 2 Department of Neonatology, La Paz University Hospital, Madrid, Spain; 3 Hospital La Paz Institute for Health Research-IdIPAZ, Madrid, Spain; 4 Division of Neonatology, Department of Pediatrics, Faculty of Medicine, Gazi University, Ankara, Turkey; 5 Division of Neonatology, Amsterdam University Medical Centers, Amsterdam, The Netherlands; 6 Department of Neonatology, Women and Newborn Hospital, University Teaching Hospitals, Lusaka, Zambia; 7 Brighton and Sussex Medical School, and Department of Neonatology, University Hospitals Sussex, Brighton and Hove, UK; 8 Division of Neonatology, Iuliu Hatieganu University of Medicine and Pharmacy, Cluj-Napoca-Napoca, Romania

**Keywords:** Family integrated care, Parent empowerment, Family-centred rounds, Neonates, Stress, Stepped wedge cluster controlled trial

## Abstract

**Background:**

Family Integrated Care (FICare) has demonstrated positive outcomes for sick neonates and has alleviated the psychological burden faced by families. FICare involves structured training for professionals and caregivers along with the provision of resources to offer physical and psychological support to parents. However, FICare implementation has been primarily limited to developed countries. It remains crucial to assess the scalability of this model in overcoming social-cultural barriers and conduct a cost-effectiveness analysis. The RISEinFAMILY project aims to develop an adapted FICare model that can serve as the international standard for neonatal care, accommodating various cultural, architectural, and socio-economic contexts.

**Methods:**

RISEinFAMILY is a pluri-cultural, stepped wedge cluster controlled trial conducted in Spain, Netherlands, the UK, Romania, Turkey, and Zambia. Eligible participants include infant-family dyads admitted to the Neonatal Intensive Care Unit (NICU) requiring specialised neonatal care for a minimum expected duration of 7 days, provided there are no comprehension barriers. Notably, this study will incorporate a value of implementation analysis on FICare, which can inform policy decisions regarding investment in implementation activities, even in situations with diverse data.

**Discussion:**

This study aims to evaluate the scalability and adaptation of FICare across a broader range of geographical and sociocultural contexts and address its sustainability. Furthermore, it seeks to compare the RISEinFAMILY model with standard care, examining differences in short-term newborn outcomes, family mental health, and professional satisfaction.

**Trial registration:**

ClinicalTrials.gov NCT06087666. Registered on 17 October 2023. Protocol version: 19 December 2022; version 2.2.

**Supplementary Information:**

The online version contains supplementary material available at 10.1186/s13063-024-08043-7.

## Introduction

### Background and rationale {6a}

Family Integrated Care (FICare) promotes the active participation of family members in providing specialised care to their sick newborn baby admitted to the neonatal intensive care unit (NICU). This change in paradigm in neonatal care, initially developed and implemented at Mount Sinai Hospital in Toronto in the stable preterm infant, has shown promising results in improving health outcomes in this population. The first pilot study conducted by O’Brien et al. demonstrated that parental involvement in direct care led to better weight gain, increased breastfeeding rates, and reduced stress and anxiety levels among parents of preterm infants [1].

These findings were further supported in a cluster-randomised controlled trial [2], as well as in subsequent studies highlighting the positive effects of the model in accelerating maturation processes [3] (earlier full enteral nutrition [3, 4]) shorter duration of mechanical ventilation and hospital length of stay [5–9], decreased rates of late-onset sepsis [5, 6], and improvements in neurobehaviour at 18 months [7]. Recently, Moreno-Sanz [8] described the successful adaptation and implementation of FICare policies also in the unstable, critically ill premature infant and the high-risk neonate with complex medical or surgical conditions, suggesting the potential for generalising the FICare model as the standard of care in NICUs.

However, the FICare model has only been tested up to now in high-income, developed countries. FICare relies on structured professional and caregiver training and parental physical and psychological support, which implies the provision of resources. Yet, the scalability of the model to overcome social-cultural barriers and cost-effectiveness analysis has not been tested.

RISEinFAMILY is an international consortium that has been designed for adapting and implementing FICare in different regions of Europe and Africa. The expansion of FICare to other NICUs and countries necessitates further research to identify potential constraints that may limit its adoption, particularly in low- and middle-income countries with different sociocultural contexts. Understanding these barriers and developing strategies to address them will be crucial in realising the full potential of FICare and ensuring its successful implementation globally.

### Objectives {7}

The aim of the RISEinFAMILY project is to develop a FICare model adapted to different cultural, architectural and socio-economic contexts as the new international standard for neonatal care and provide sound data on its sustainability based on a cost-effectiveness analysis.

The adapted FiCare model (herein after RISEinFAMILY model) will include a wider geographical and sociocultural diversity and 2 implementation levels (basic and advanced) and will try to demonstrate its superiority, against routine current provision of care, in infants’ clinical outcomes, parental mental health and professional’s self-care and satisfaction.

### Trial design {8}

**Fig. 1:**
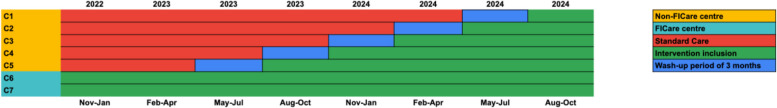
Schematic illustration of stepped wedge cluster trial

This quality improvement project is designed as an international, multi-centre, pluri-cultural prospective stepped-wedge cluster controlled trial. Five non-FICare-experienced NICUs from Netherlands, Turkey, Romania, the UK and Zambia (sites 1–5) and two clinical sites who have recently implemented FICare from Spain and Netherlands (sites 6 and 7) are expected to be recruiting infants into this trial that will include at least 552 babies during a 32-month period (see Fig. 1). The stepped wedge cluster design was selected due to the nature of the intervention, which involves changes to unit-level provision of care and interaction between participants, with a risk of cross-contamination.

## Methods: participants, interventions and outcomes

### Study setting {9}

Six academic hospitals from Netherlands, Turkey, Romania, the UK, Zambia and Spain (sites 1–6) and one non-academic hospital (site 7) will collect data for this study. The list of the study sites of the RISEinFAMILY consortium, H2020-MSCA-RISE-2020, can be found here https://cordis.europa.eu/project/id/101007922 [9].

### Eligibility criteria {10}

We will include infants born with birth weight at or below 1500 g or gestational age at or below 34 weeks, any other peri-neonatal condition anticipating NICU specialised care with an admission for at least 7 days and the decision to provide full life support. Further, we will include parents if they are willing to spend at least 6 h per day at NICU or committed to attending educational sessions, an active involvement in care of their infant for at least a 7-day period, not to have an intellectual or language barriers to understanding, at least one primary caregiver is involved in the training and signed informed consent. We will exclude infants with the decision not to provide full life support, critical illness unlikely to survive or scheduled for early transfer to another non-FICare hospital. We will exclude parents if they have an intellectual handicap that makes learning-understanding difficult, communication cannot be established even with a translator, have mental psychiatric problems or under legal supervision, or guardianship of social services, and finally, lack of parental signed informed consent.

### Who will take informed consent? {26a}

Written documentation of informed consent is obtained before initiating the intervention following approval by the Ethics Committee. The templates for these forms have been developed in collaboration with parent groups and the European Foundation for the Care of Newborn Infants (EFCNI) (see Additional file 2). Each clinical site will adapt the templates for submission to the regulatory authorities in their respective countries. Upon meeting the inclusion criteria, parents or patient’s legal representative will receive a detailed parent information letter, providing extensive information about the trial and intervention. They have the freedom to withdraw their child from the trial at any stage and for any reason, without impacting their child’s ongoing treatment. The investigator or research nurse will obtain separate consent for data collection concerning both the mother and the newborn. Also, a separate consent form for healthcare professionals is developed. Consent forms will be included as part of the process.

### Additional consent provisions for collection and use of participant data and biological specimens {26b}

This trial does not involve collecting biological specimens for storage.

### Interventions

#### Explanation for the choice of comparators {6b}

A cohort of patients, born at clinical sites 1 to 5 from the beginning of the study to the time assigned to start the intervention, will be assigned to the control group, representing standard neonatal care.

#### Intervention description {11a}

The study will start at the same time at all participating sites that will continue to provide routine clinical care according to their current policies. Prospective data gathering will be accomplished.

A start point for the experimental intervention (3, 6, 9, or 12 months from the start of the study) will be assigned for each clinical site (1 to 5) taking into account the expected time internally needed to overcome all logistics and regulatory issues with a potential impact on FICare implementation. A 3-month wash-out period will be used for the local training process and assessment of site readiness, while enrolment will be halted. After that, recruiting will continue, and the experimental intervention will be started. Clinical sites 6 and 7 will run the experimental intervention from the start of the study, without pauses.

##### Training and certification

The RISEinFAMILY training programme is divided into two curricula: for healthcare professionals/staff (Training the trainers) and for families (Education of caregivers), the latter being categorised into two intervention levels (basic and advanced). The following modules are identified as the minimum training contents to be delivered to foster RISEinFAMILY:

- *Training the trainers:* (1) understanding the boundaries of the FICare model; how to promote FICare among families; (2) psychosocial needs of families (resilience, stress and anxiety, or mourning); communication skills (assertive communication); (3) how to involve families in NICU (safe conduct in NICU environment, attachment and bonding, how to do family-centred medical rounds); (4) professional self-care (burnout, compassion fatigue).
- *Education of caregivers:* (1) comprehensive description of the FICare model (the strengths and training methodology) and the functional and architectural structure of the NICU; (2) family self-care (stress and anxiety, resilience, or mourning); (3) learning about infants’ neurobehavior, stress and pain; (4) taking part in baby care (basic level), where parents will be “professionalised” as to become the first line care provider of their children; (5) taking part in baby care (advanced level), specific task’s training for infants who require even more specialist care; (6) parents will be prepared for home; a map of the social resources available at the local setting will be provided.

A teacher education system will be used for staff training. Mentor-assigned trainee groups and the calendar for meetings will be defined by site co-ordinators. The procedure will include face-to-face meetings, e-learning tools or a combination of methods adapted to centre facilities and organisational features, in order to accomplish the programme and procedures dissemination among 90% of the NICU staff.

The family training process relays on three cornerstones: (a) individualised theoretical and practical learning by tasks through face-to-face sessions at the cot-side, following an individualised teaching plan adjusted to the baby’s clinical condition and the wish of the parents; (b) family workshops, held as presential, online or hybrid format, that intend to gather parents during 45-min open sessions on relevant topics about the learning contents, to express their doubts and concerns, as well as to share their experiences with other families; (c) registry of teaching activities and task certification once proficiency is fully accredited in a given task, and the family caregiver is allowed to do this task autonomously.

#### Criteria for discontinuing or modifying allocated interventions {11b}

The criteria for discontinuing or modifying allocated interventions for a given trial participant include parents’ request for temporary suspension due to personal reasons or the patient’s worsening condition leading to their desire to withdraw from active participation.

#### Strategies to improve adherence to interventions {11c}

Implementing family-centred rounds and educational workshops allows for continuous evaluation of families’ learning process, fostering better adherence to intervention protocols.

#### Relevant concomitant care permitted or prohibited during the trial {11d}

Implementing the RISEinFAMILY model will focus on the active participation of family members in providing specialised care to their sick newborn baby admitted to the neonatal intensive care unit; this fact will not alter the usual care treatment pathways (including the use of any medication) that will continue for both trial arms.

#### Provisions for post-trial care {30}

 Implementing the RISEinFAMILY model will focus on the active participation of family members in providing specialised care to their sick newborn baby admitted to the neonatal intensive care unit, it will not alter the usual care treatment pathways (including use of any medication) and these will continue for both trial arms.

### Outcomes {12}

The study’s primary outcomes are as follows: (a) RISEinFAMILY implementation: proportion of families completing basic and advanced training levels (observed vs expected), average time to complete basic and advanced training levels (observed vs expected) and average time of kangaroo care per day (hours); (b) short-term health infant’s outcomes: proportion of high-risk infants achieving and maintaining adequate growth patterns during NICU admission, defined according to Patel’s method [10, 11].

The study’s secondary outcomes are as follows: reported adverse event rate per 1000 patients/day, feeding patterns and major morbidities at 36 weeks postmenstrual age (PMA) or discharge, nosocomial infection [12], necrotising enterocolitis (Bell’s > stage 2), moderate-severe brain injury (worst cranial ultrasound) [13], parental needs, empowerment and psychological health (stress [14], anxiety and depression [15, 16], self-efficacy [17], bonding [18], resilience [19]), professional’s self-care and satisfaction (anxiety and depression [15], burnout [20], post-traumatic stress [21], work and well-being [20]).

The study’s exploratory outcomes are as follows: economic impact (level of post-implementation utilisation of FICare, resources and costs associated with FICare implementation, cost-effectiveness estimates compared to the current care, expected value of current implementation and expected value of perfect implementation); mid-term infant’s general health (growth pattern during the first 12 months from birth, use of health system facilities after discharge); long-term neurodevelopment (survival without neurodevelopmental disabilities).

Specific items and test batteries are stated in Additional file 1.

#### Questionnaires for parents

Abbreviated Parental Stressor Scale for Pediatric Intensive Care Unit (A-PSS:PICU) [22]: This scale, based on the PSS:PICU [14] includes 7 items to assess parental stress caused by the PICU environment. It has two factors (stress due to the child’s condition and stress related to PICU’s staff) and adequate internal consistency (*α* = 0.80). In this article, we have adapted the items to the NICU context.

Generalized Anxiety Disorder (GAD-7) questionnaire [23]: This is a seven-item questionnaire designed to assess the patient’s anxiety symptoms during the previous 2 weeks. It showed excellent internal consistency (*α* = 0.92) and test–retest reliability (intraclass correlation = 0.83). At a cut point of 10 showed the greatest sensitivity and specificity.

Edinburgh Postnatal Depression Scale (EDPS) [24]: This is a 10-item scale to measure depressive symptoms during the perinatal period. It showed excellent internal consistency (*α* = 0.91) and test–retest reliability (Spearman’s rho rank correlation = 0.90). The cutoff score of ≥ 13 indicates an elevated risk of depression.

The Perceived Maternal Parenting Self-Efficacy (PMP S-E) [17]: This is a 20-item questionnaire to measure maternal parenting self-efficacy, especially of hospitalised preterm neonates. It showed excellent internal consistency (*α* = 0.91) and test–retest reliability (Spearman’s rho rank correlation = 0.96).

The Postpartum Bonding Questionnaire (PBQ) [18]: It comprises 25 items with a 5-point Likert-type response scale. It measures the presence of bonding difficulties and has four subscales: affected bonding, rejection and hatred, anxiety about baby care, and risk of abuse. It provides a total score of the quality of bonding. It showed excellent internal consistency (*α* = 0.90) and test–retest reliability for the total scale (Spearman’s rho rank correlation = 0.96), and its subscales (Spearman’s rho rank correlation ranging from 0.77 to 0.95).

Brief Resilience Scale (BRS) [25] is a 6-item self-report scale that was designed to assess resilience as the ability to bounce back from stress. It has proved adequate reliability, shown adequate internal consistency (*α* ranging from 0.80 to 0.90) and test–retest reliability (*r* = 0.62–0.69) and validity.

Perceived social support scale: This is an ad hoc questionnaire designed to measure the degree to which parents were satisfied with the social support they were receiving now from their partner, family, friends and other close people different from family and healthcare staff.

Posttraumatic Stress Disorder-8 (PTSD-8) inventory [21] is an 8-item scale for screening for probable PTSD. The scale originates from the Harvard Trauma Questionnaire (HTQ) [26]. It consists of eight items rated on a 4-point severity scale. Positive screening requires a concurrent rating of at least one item from each symptom cluster (intrusion, avoidance, hypervigilance) with a score of three or higher. Its scores have shown adequate internal consistency (*α* = 0.83–0.85).

The 9-item Shared Decision Making Questionnaire (SDM-Q-9) [27] is a 9-item measure of the decisional process in medical encounters from the patients’ perspective. It has good reliability (*α* = 0.94) and high face and structural validity.

Posttraumatic Growth Inventory Short Form (PTGI-SF) [28] is based on the 21-item original version by Tedeschi and Calhoun [29], designed to measure the positive legacy of trauma. The PTGI-SF contains 10 items to measure five domains: greater appreciation of life, improved relationships with others, greater personal strength, recognition of new possibilities in one’s life course and spiritual or religious growth. It has shown adequate internal consistency (*α* ranging from 0.84 to 0.90) and validity. In order to make sure that the PTG that parents reported was a consequence to their child’s admission to the NICU; instead of asking about responses “as a result of my crisis”, we asked about responses “result of your child’s admission to the NICU”.

#### Questionnaires for healthcare professionals

The Four-Item Patient Health: PHQ-4 [15] is a valid ultra-brief tool for detecting both anxiety and depressive disorders. It includes two items that measure depression used in the PHQ-8 and two items that measure anxiety used in the 7-item Generalised Anxiety Disorder (GAD-7) scale. Each item is rated on a 4-point Likert scale. The PHQ-4 sum score is classified as none (0–2), mild (3–5), moderate (6–8) and severe (9–12) symptoms of general/unspecific anxiety and depression. Its scores have shown adequate construct and factorial validity.

Perception About Parental Participation: This is an ad hoc questionnaire designed for the purpose of this study. It includes 12 items on a 6-point Likert scale to measure healthcare workers’ perception about parental participation in the NICU. It measured to which degree professionals considered that (1) parental presence during medical procedures, (2) parental presence during medical rounds and decision-making processes, (3) open-door policies in the NICU, and (4) parental participation in their children’s care was beneficial for parents, the baby, and professionals.

Maslach Burnout Inventory (MBI) [30] is a 22-item questionnaire that assesses the frequency of occurrence of different feelings in relation to their job in the last week in a 7-point Likert scale. It contains three dimensions: emotional exhaustion (EE), depersonalisation (DP) and personal achievement (PA). A meta-analysis has shown an average internal consistency (Cronbach’s *α*) of 0.88, 0.71, and 0.78, respectively for each dimension [31]. Cutoff scores for EE are between 15 and 24 (the score is low if it is below 15 and high if it is up to 24), for DP between 4 and 9 and for PA between 33 and 39.

UNWES-9 Work & Well-being Survey (UWES) [32] is a 9-item questionnaire to measure work engagement. It includes three dimensions to measure vigour, dedication, and absorption. It has shown adequate factorial validity and internal consistency (Cronbach’s *α* for the total scale between 0.85 and 0.92).

9-item Shared Decision Making Questionnaire-version for physicians (SDM-Q-Doc 9 items) [33]: This is a 9-item measure of the decisional process in medical encounters from the physicians’ perspective. It has shown adequate internal consistency (Cronbach’s *α* of 0.88). Factor analysis confirmed a one-dimensional structure.

### Participant timeline {13}

See Table 1.

**Table 1:** Activity scheme

	**Screening**	**Enrolment**	**Intervention**	**End of intervention**	**Short-term endpoint assessment**	**Mid-term endpoint assessment**	**Long-term endpoint assessment**
**Date**	x	x	x	x	x		
**Informed consent document**	x						
**Signed informed consent document**		x					
**Eligibility**	x						
**Demographic and perinatal data**		x					
**Parental data**		x					
**NICU facilities**		x					
**FICare profile**		x					
**Day of life and PMA**		x		x	x	x	x
**Neonatal outcomes**					x		
**Task on training**			x	x			
**Kangaroo (hours/week)**			x				
**Weight, length, head circumference**		x	x	x	x	x	x
**Nutrition**				x	x	x	
**Maturation skills**				x	x		
**Adverse events**			x	x	x		
**Reason for early discontinuation**				x			
**General health**						x	x
**Neurodevelopment**							x
**Economic impact**^a^			x	x	x		x
**Parental questionnaires**		x			x	x	
**Staff questionnaires**^b^							

X indicates mandatory procedures that should be entered into the eCRF

*NICU* neonatal intensive care unit, *FICare* Family Integrated Care, *PMA* postmenstrual age

^a^Economic impact on newborns, carers and staff as described in Additional file 1

^b^2 measurement points: first time before FICare implementation at his/her institution; second time at least 3 months after FICare implementation at his/her institution. In FICare expert centre only one time

### Sample size {14}

No previous study has assessed the effect of FICare intervention on growth velocity expressed in g/kg per day. Therefore, for sample size calculation in the RISEinFAMILY project, we will follow one of the most recent studies evaluating the effect of individualised breast milk fortification on weight gain expressed in the same units [34]. In this study, based on their own data obtained in a pilot and other literature data, the authors assumed 1.8 ± 3.1 g/kg/d as a reasonable and clinically meaningful difference in weight gain. With a total *N* of 103 (experimental group *N* = 52, control group *N* = 51), they found a difference in the weight gain of 1.9 g/kg/d and a standard deviation of 2.5 g/kg/d.

Using this information, we calculated the minimum size for the intervention (FICare) and the control (standard care) group in the RISEinFAMILY study. Our null hypothesis is no impact of FICare and, as a consequence, the mean of the weight gain in both the intervention and control groups will be the same. Our alternative hypothesis is a difference in the mean weight gain between FICare and the control group of at least 1.9 g/kg/d [34]. We impose a type I error (probability of rejecting the null hypothesis when it is true) of 5% (*α* = 0.05), and a type II error (probability of not rejecting the null hypothesis when it is false) also of 5% (> 0.05), which provides a statistical power of 95%. The minimum sample size for intervention and control groups is given by at least 46 children in the intervention (FICare) and other 46 children in the control (standard care) group.

### Recruitment {15}

This is a stressful time for parents and families. Coping with all the information provided at this early stage is difficult for parents. For this reason, a strategy for recruitment is in place to avoid overloading parents with information during the critical period after birth when they are most vulnerable:

- Prior to any enrolment, information about the RISEinFAMILY project will be made public in participating hospitals through leaflets, posters, and the use of modern technology.
- Whenever possible, a summary with preliminary information about the programme will be distributed to parents of potential candidates antenatally, in order to provide them with more time to think about joining the study if the baby and family become eligible after birth. Contact information for the local quality improvement team will be provided.
- Whenever possible, parents will give written informed consent antenatally after an explanation of the aims, methods, benefits and potential hazards of the programme. The implications of the programme’s intervention to the neonates and families enrolled should be clearly explained by the local research team as part of the informed consent process. Perinatal committees are the ideal forum to comment on potential candidates. These committees are the forum where complicated pregnancies are discussed among obstetricians and neonatologists. Appointment with eligible families can be scheduled according to the information gathered in these meetings, allowing a full explanation of the RISEinFAMILY project before delivery.
- Parents that have not yet given written informed consent for the study will be approached as soon as possible after the baby is born and admitted to the NICU and provided with the relevant parental information.

## Assignment of interventions: allocation

### Sequence generation {16a}

In this non-randomized study, the allocation sequence is not generated using computer-generated random numbers. Instead, the allocation is based on considerations aimed at achieving an equitable distribution of patients between the control and intervention groups.

### Concealment mechanism {16b}

For the study at clinical sites 1-5, the initiation involves a control phase where standard neonatal care procedures are followed. Subsequently, during the wash-up period, the implementation of the intervention begins. The concealment mechanism is considered not applicable in this context.

### Implementation {16c}

The study design adopts a non-randomized approach, with an initial period during which clusters 1-5 are not exposed to the intervention. At regular intervals referred to as “steps”, clusters transition from the control to the intervention, leading to a systematic exposure until all clusters undergo the RISEinFAMILY project: a study protocol for the integration of families at Neonatal Intensive Care Units (NICUs) to empower them as primary caregivers, 20-11-2023 14-02-2024 page 12 of 21 crossover. Towards the study's conclusion, a phase ensues where all clusters uniformly experience the intervention. Data collection persists throughout, allowing each cluster to contribute observations during both control and intervention periods.

## Assignment of interventions: blinding

### Who will be blinded {17a}

Given the logistical and regulatory challenges in implementing randomization at clinical sites 1 to 5, blinding is not feasible. This is due to variations in hospital size and the internal time required to address logistical and regulatory issues. The primary objective remains the achievement of an equitable distribution of patients between the control and intervention groups. Consequently, hospitals and healthcare professionals involved in this trial will not be blinded for the assigned trial protocol.

### Procedure for unblinding if needed {17b}

The design is open-label with only outcome assessors being blinded so unblinding will not occur.

## Data collection and management

### Plans for assessment and collection of outcomes {18a}

Data will be collected by researchers at participating hospitals and stored anonymously in a digital database (Research Electronic Data Capture; REDcap). Data will be stored in accordance with guidelines issued by the Spanish Data Protection Agency. Independent monitors will perform source data verification and assess the performance of trial procedures at least once a year at each site. FIBHULP will be responsible for the data management and storage of the study data. The veracity of the data will be ensured through the training of the professionals in charge of the REDCap database. In addition, a database user manual has been included in the study folder accessible to all researchers.

There will be several time points for data entry: screening, enrolment, weekly visit up to discharge to home or transfer to a non-FICare centre, short-term clinical outcome assessment, mid-term clinical outcome assessment and long-term clinical outcome assessment. If the infant in the meantime has been discharged to a step-down unit, data should be sought from the specific unit. If this is not possible, data should be used until the date of discharge to the step-down unit.

### Plans to promote participant retention and complete follow-up {18b}

Intermediate trial report is planned to confirm that clinical sites are aligned with the estimated recruitment rates, or to refine. This will facilitate reaching the calculated sample size.

### Data management {19}

FIBHULP will be responsible for the data management and storage of the study data (on a database) including:

- *Data storage and backup*: The study server running the database is regularly maintained by the manufacturer. Twice a day a backup of the database with pseudonymised study data and the application data is automatically generated. Further storage of the dump files in an additional secure area and protected by a password will be issued. These backups can be used to restore the data and the application for electronic data capture on another server within a short time period.
- *Data validation*: Data will be validated according to the data validation plan.
- *Data coding:* medical history, adverse events (when applicable please refer to the assessment of safety section of the protocol) and any abnormality obtained on any study test result will be coded using MedDRA and concomitant treatments using WHO-DD. The codification procedure is described in a specific manual.

After conducting all data validation and the final review, the study database will be considered as completed and its containing data as reliable. At this moment, the study database will be closed and transferred to the Biostatisticians team for data analyses.

At the end of the study, a copy of the site-specific records will be provided to each principal investigator.

### Confidentiality {27}

The study staff will ensure that the participant´s anonymity is maintained. The participants will be identified only by a participant code on the eCRF and any electronic database. All documents will be stored securely and only accessible by study staff and authorised personnel. Applicable regulations for storage, transmittal and disclosure of patient information will be followed at all times. The study will comply with the Data Protection Legislation in each country. Following formal admission to the study, patient data will be recorded in the hospital case record in the usual way including the circumstances of their entry to the study. Additionally, data will be held in case report forms (eCRF). These files will be identified by a study code, date of birth and participant code only. Representatives from the Sponsor and from the regulatory authorities will be given access to the records that relate to the study. They will have full access to the anonymous eCRFs for the purposes of data validation. Results of the study may be communicated at scientific meetings and will contribute to the scientific literature. At no time, will this be done in such a way that an individual patient may be identified.

### Plans for collection, laboratory evaluation and storage of biological specimens for genetic or molecular analysis in this trial/future use {33}

See above 26b; there will be no biological specimens collected.

## Statistical methods

### Statistical methods for primary and secondary outcomes {20a}

The primary analysis will be conducted in the intention-to-treat (ITT) population including all enrolled patient-family dyads who received at least one task training according to protocol procedures.

Descriptive statistics will be recorded for each group with mean, median and standard deviation for numerical variables and absolute and relative frequencies for categorical variables. 95% confidence intervals (CIs) will be provided. Comparisons between control and intervention groups will be performed using chi-square tests for categorical variables, analysis of variance for normally distributed continuous variables, and Mann–Whitney or Kruskal–Wallis tests for non-normally distributed continuous variables. Through a mixed-methods research approach and techniques both quantitative and qualitative, the following impacts will be evaluated: (a) effect of intervention: the control and the intervention group dataset from the non-FICare-experienced centres will be compared; (b) effect of “expertise” on outcomes: comparisons will be conducted between datasets from already trained FICare centres and those from the non-FICare-experienced centres.

Socio-economic sustainability: Health economics analysis based on the value of implementation framework will be conducted to assess the cost-effectiveness of RISEinFAMILY implementation strategies. The difference between the total net benefit of perfect adoption and the total net benefit of current implementation will generate the expected value of perfect implementation (positive values would characterise cost-effective implementation strategies). The analysis will also allow identifying differences in delivering RISEinFAMILY in different countries/health care settings.

Psychological assessment of experiences collected by either the NICUs staff and the families enrolled in the pilots will be conducted. Gender differences will be explored in relation to socioeconomic and cultural background.

For our primary hypothesis, we will compare infant standardised weight gain velocity between the two groups over the 4-week study period using generalised linear mixed modelling (GLMM). For our primary outcome, we will compare infant standardised weight gain between the two groups over the study period defined as the change in Patel’s growth pattern measured weekly from enrolment until day 28 post-enrolment, testing an interaction term between group and post-baseline weight measurements. We will adjust for additional covariates using a hybrid approach, forcing in known confounders of gestational age and study site and using backward stepwise selection to retain covariates that contributed *p* < 0.1 to the final model from potential confounders. Additionally, we will conduct sensitivity analyses by adding in weight measured at discharge.

### Interim analyses {21b}

No detrimental problems to the study participants are anticipated. In addition, conflicts of interest within the decision-making process during the trial’s execution, specifically concerning the reallocation of resources to address unmet needs, are expected. Therefore, no interim analyses are planned.

### Methods for additional analyses (e.g. subgroup analyses) {20b}

Important prognostic variables are centre, sex and gestational age, or main neonatal diagnosis. These will be used as additional independent variables in key secondary logistic regression models for secondary subgroup analysis.

Further subgroup analysis will be conducted in line with the primary analysis, where the endpoint is dichotomous. For continuous endpoints, similar modelling strategies will be used, but instead of logistic regression linear regression models will be used.

The cost-effectiveness analysis of RISEinFAMILY implementation will be carried out using recommended methods [35, 36]. A decision-analytical [37] will be developed and populated with costs and effectiveness data from the pilot studies. For each RISEinFAMILY implementation site, the cost-effectiveness of the intervention will be estimated. The cost-effectiveness and the value of implementation analyses will be conducted for the seven neonatal units involved in the study. The mean net monetary benefit per participant will be calculated using the health care system willingness-to-pay threshold corresponding to the current cost of neonatal care in the NICU.

### Methods in analysis to handle protocol non-adherence and any statistical methods to handle missing data {20c}

Incomplete data will be addressed using multiple imputation chain equations (mice), taking into account clustering adjustments. Rubin’s rules will be employed to combine the findings from various imputed datasets. Following the recommendations of the Panel on Handling Missing Data in Clinical Trials, sensitivity analyses will be conducted with diverse strategies for handling missing data, incorporating adjustments for clustering. This will encompass considering missing data scenarios such as completely at random, missing at random and missing not at random.

### Plans to give access to the full protocol, participant-level data and statistical code {31c}

The datasets analysed during the current study and statistical code are available from the corresponding author on reasonable request, as is the full protocol.

## Oversight and monitoring

### Composition of the coordinating centre and trial steering committee {5d}

In the trial, La Paz University Hospital served as the coordinating centre and trial steering committee. It comprised the chief investigator, technical coordinator, financial officer and legal officer. Additionally, each participating centre had its own chief investigator overseeing the trial’s day-to-day operations.

### Composition of the data monitoring committee, its role and reporting structure {21a}

Due to the nature of the intervention, which does not concern a medical drug and does not propose extra risk to the infants and parents, the implementation of a Data Safety Monitoring Board is not deemed necessary.

### Adverse event reporting and harms {22}

Considering the nature of the intervention and the characteristics of the patients who may present complications intrinsically to their biological or medical condition, serious adverse events will not be individually reported but will be part of the data collected as part of the assessment of benefits and harms.

### Frequency and plans for auditing trial conduct {23}

The electronic data entry system provides an audit trail, allowing identified and authorised users to remotely store data in the eCRFs so that all data entries and changes are done by sites in the central database are automatically and chronologically recorded.

### Plans for communicating important protocol amendments to relevant parties (e.g. trial participants, ethical committees) {25}

Our communication plans for important protocol modifications prioritise timely and direct dissemination of information to all relevant parties. This includes investigators, Research Ethics Committees/Institutional Review Boards (REC/IRBs) and trial registries. Through efficient channels of communication, we will ensure that updates are promptly shared, allowing all stakeholders to stay informed and make necessary adjustments or decisions accordingly.

### Dissemination plans {31a}

The trial findings will undergo consideration for publication and presentation at scientific symposia or congresses. Authorship will adhere to the guidelines established by the International Committee of Medical Journal Editors (http://www.icmje.org). As participant data will be recorded anonymously, utmost care will be taken to ensure participant privacy. The results obtained from the trial will contribute to the enhancement of existing guidelines and enable the publication of new ones.

In addition to traditional academic avenues, we acknowledge the importance of broader communication. Therefore, we will develop a lay summary of the trial findings to share with all participating families, ensuring accessibility and transparency. Additionally, we will explore the possibility of conducting stakeholder workshops to engage with relevant groups and gather valuable perspectives.

## Discussion

This international multicenter trial focusing on FICare marks a significant milestone in the field as it explores the scalability of FICare across diverse geographical and sociocultural contexts. This comprehensive research study protocol aims to fill gaps in existing literature and advance knowledge in the field by outlining the study design, methodology, and objectives. The trial employs a stepped wedge cluster controlled trial design to provide strong evidence on the impact of FICare. This type of study design not only offers practical and ethical benefits but also enhances generalisability by accounting for confounding effects from factors such as hospital culture, policies, architecture, and size. The analysis also considers the interaction of time, acknowledging the potential growth of parental involvement in neonatal care practices.

Key outcomes of interest include short-term newborn health, parental mental health, and healthcare professional satisfaction. The collaborative co-design process strengthens the study by involving various stakeholders and ensuring the relevance and applicability of the findings. Additionally, the study examines economic outcomes in different developmental countries to provide insights into the cost-effectiveness of implementing FICare.

While a limitation of this study is the lack of blinding for participants and researchers, it is anticipated to have minimal impact at the individual patient level, as researchers cannot manipulate medical facts or influence parental responses. Although there is a potential bias from non-blinding healthcare professionals who may have pre-existing support for FICare, this bias is expected to be insignificant. Objective outcomes at the cluster level, such as cost-effectiveness, are not anticipated to be affected by bias.

It is important to consider the potential variations in the execution of the intervention across different hospitals, particularly regarding the human factors involved, such as the communication and collaborative skills of healthcare professionals. Ultimately, the robust evidence generated from this research will support the implementation of an international FICare package, regardless of architectural, cultural or economic differences. This will lead to improved neonatal care and enhanced outcomes for families and healthcare professionals. The study results are expected to contribute to the existing evidence base and inform future interventions and clinical practices in neonatal care worldwide.

## Trial status

This manuscript is based on the trial protocol “Integrating families at neonatal intensive care units for empowering them as primary caregivers (RISEinFAMILY)”, version 2.2, December 2022. The project began in September 2021, and patient enrolment commenced in February 2023. The study is in the stage of the main study. The registration number of the study in ClinicalTrials.gov is NCT06087666. The whole study is scheduled to be completed by the end of December, 2025.

### Supplementary Information

**Supplementary Material 1. **

**Supplementary Material 2. **

## Data Availability

FIBHULP will guard the research data through a web-based electronic clinical trials data management system. All multicentre data analysis shall be conducted together with FIBHULP. For single site data analysis: All RISEinFAMILY site investigators will receive, upon request to FIBHULP, a file of all data on all subjects enrolled at their institutions in the RISEinFAMILY project after database closure and finalising of the clinical trial report. FIBHULP will not in all instances be able to provide data analysis for single institution publications, though it will be happy to provide advice and guidance. Direct access to source data will be granted to authorise representatives from the Sponsor or delegated organisation, host institution, Ethic Committees and Regulatory Authorities study-related for monitoring, audits and inspections.

## Abbreviations

AR: Adverse reaction
CI: Confidence Interval
CPC: Clinical Publication Committee
EDC: Electronic Data Capture
eCRF: Electronic Case Report Form
EFCNI: European Foundation for the Care of Newborn Infants
EPDS: Edinburgh Postnatal Depression Scale
ES: Spain
FIBHULP: Fundación para la Investigación Biomédica del Hospital Universitario La Paz (Biomedic Investigation Foundation of La Paz University Hospital)
FiCARE: Family Integrated Care
GCP: Good Clinical Practice
GU: Guzi University
HULP: La Paz University Hospital
ITT: Intention-to-treat
MedDRA: Medical Dictionary for Regulatory Activities
NICU: Neonatal intensive care unit
NL: Netherlands
OLVG: Onze Lieve Vrouwe Gasthuis
OR: Odds ratio
PI: Principal Investigator
PMA: Postmenstrual age
PSS-NICU scale: Pediatric Stress Scale-NICU scale
ProQOL scale: Pro Quality of Life scale
RO: Romania
SERMAS: Servicio Madrileño de Salud (Madrid, Spain)
TR: Turkey
UCICEC-HULP: Central Research and Clinical Trials Unit at La Paz University Hospital
UK: United Kingdom
UHS: University Hospitals Sussex
ZM: Zambia Midwives Association
ZSM: University of Zambia School of Medicine
