# Supplementary material for: RISEinFAMILY project: the integration of families at neonatal intensive care units (NICUs) to empower them as primary caregivers: study protocol for a stepped wedge cluster controlled trial
Source: Trials. 2024 Apr 10;25:248. doi: 10.1186/s13063-024-08043-7 (PMC11005221; doi:10.1186/s13063-024-08043-7)
Supplement: Supplementary file 1 — Supplementary Material 1. [file 13063_2024_8043_MOESM1_ESM.docx]

# Additional file 1

**Table 2. Neonatal follow-up**

| **Time** | **Outcome Measure** |
| --- | --- |
| T=0 (at enrollment/admission) | Obtain parental consent |
| During hospital stay 1x/week | Anthropometric data |
|  | Growth parameters |
|  | Breastfeeding/feeding parameters |
|  | Parental presence |
| T=1 (at discharge from hospital) | Growth parameters |
|  | Feeding regimen/breastfeeding |
|  | Duration of hospital stay |
|  | Sepsis |
|  | Respiratory support |
|  | NEC (bell stage 2 or more) |
|  | Bronchopulmonary dysplasia |
|  | Retinopathy of Prematurity |
|  | ECMO therapy |
|  | Reported adverse events |
| T=2 (CA 3-6 months) | Growth parameters |
|  | Feeding regimen/breastfeeding |
|  | (Re)admission to hospital |
|  | Frequentation of Emergency service |
| T=3 (CA 12 months) | Growth parameters |
|  | Feeding regimen |
|  | (Re)admission to hospital |
| T=4 (CA 24 months) | Growth parameters |
|  | (Re)admission to hospital |
|  | Neurodevelopmental impairment |
|  | Hearing loss |
|  | Blindness |
|  | Cerebral palsy |

t:time, CA:corrected age

**Table 3. Parental follow-up**

| **Time** | **Outcome Measure** | **Questionnaire** |
| --- | --- | --- |
| T=0 (at enrollment/admission) | Parental Stress levels | PSS:NICU |
|  | Screening for anxiety | GAD-7 |
|  | Screening for postnatal depression | EPDS |
|  | Parental self-efficacy | PMP S-E |
|  | Parent-infant bonding | PBQ |
|  | Parental resilience | BRS |
|  | Perceived social support | n.a. |
|  | Maternal obstetric data | n.a. |
|  | Parental data | n.a. |
| T=1 (at discharge from hospital) | Parental Stress levels | PSS:NICU |
|  | Screening for anxiety | GAD-7 |
|  | Screening for postnatal depression | EPDS |
|  | Parental self-efficacy | PMP S-E |
|  | Parent-infant bonding | PBQ |
|  | Parental resilience | n.a. |
|  | Post-traumatic stress in parents | PTSD-8 |
|  | Shared Decision Making | SDM-Q-9 |
| T=3 (CA 3-6 months) | Screening for anxiety | GAD-7 |
|  | Screening for postnatal depression | EPDS |
|  | Parental self-efficacy | PMP S-E |
|  | Parent-infant bonding | PBQ |
|  | Perceived social support | n.a. |
|  | Post-traumatic stress in parents | PTSD-8 |
|  | Post-traumatic Growth in parents | PTQ |
|  | Psychological support | n.a. |

t:time, CA:corrected age, n.a.: not applicable

**Table 4. Healthcare professionals**

| **Time** | **Outcome Measure** | **Questionnaire** |
| --- | --- | --- |
| T=0 (at start of the study) | Demographic data | n.a. |
|  | Work-related data | n.a. |
|  | Patiënt health | PHQ-4 |
|  | Parental participation | n.a. |
|  | Burn-out inventory | MBI-HSS |
|  | Post traumatic stress disorder | PTSD-8 |
|  | Work & well being | UWES-9 |
|  | Shared decision making | SDM-Q-9-Doc |
| T=1 (at the middle of the study) | Patient health | PHQ-4 |
|  | Parental participation | n.a. |
|  | Burn-out inventory | MBI-HSS |
|  | Post traumatic stress disorder | PTSD-8 |
|  | Work & well being | UWES-9 |
|  | Shared decision making | SDM-Q-9-Doc |
| T=2 (at the end of the study) | Patiënt health | PHQ-4 |
|  | Parental participation | n.a. |
|  | Burn-out inventory | MBI-HSS |
|  | Post traumatic stress disorder | PTSD-8 |
|  | Work & well being | UWES-9 |
|  | Shared decision making | SDM-Q-9-Doc |

t:time, CA:corrected age, n.a.: not applicable

**Table 5. Economic impact**

| **Participants** | **Outcomes** |
| --- | --- |
| Neonates | - Number of days in hospital. - Number of days on different levels of care (intensive care, high-dependency care, specialist care, normal care) - Daily cost of care per level - Hospital readmissions (number of episodes) - Frequentation of emergency service (number of episodes recorded since the last visit) |
| Carers | - Personal expenses questionnaire: siblings on care, relation to the newborn on care, average hours/day on care, overnight stay at hospital/nearby, payment for overnight stay (amount), travel to hospital for care (days/week, expenses/travel), other expenses and costs (listed), other dependents under your care, any help for dependents, additional costs for helpers, other expenses (costs), - Costs of training materials for carers (brochures, laptops, mobile devices) |
| Staff | - FICare Training Questionnaire for instructors: job title, pay range, training sessions (number attendees, duration, time spent on preparation, travel expenses associated to training, - FICare Delivery Questionnaire: job title, pay range (5 categories), training received (hours), average daily hours FICare on ward, average daily hours on FICare documentation, other FICare-related duties (time) - Cost of training materials for staff (brochures, laptops, mobile devices...) |
