# Supplementary material for: RISEinFAMILY project: the integration of families at neonatal intensive care units (NICUs) to empower them as primary caregivers: study protocol for a stepped wedge cluster controlled trial
Source: Trials. 2024 Apr 10;25:248. doi: 10.1186/s13063-024-08043-7 (PMC11005221; doi:10.1186/s13063-024-08043-7)
Supplement: Supplementary file 2 — Supplementary Material 2. [file 13063_2024_8043_MOESM2_ESM.docx]

**Additional file 2**

**Informed consent materials {32}**

**INFORMATION SHEET FOR PARENTS OR GUARDIANS**

**Title of the study: Integrating families at neonatal intensive care units for empowering them as primary caregivers (RISEinFAMILY)**

**Introduction**

Dear parents,

Congratulations on the birth of your baby. You are receiving this information sheet because your baby has been admitted to our neonatal unit. We understand that this is a very stressful time for you and it may be difficult for you to read this information at this time. This leaflet provides you with information about the RISEinFAMILY project.In this project we would like to offer parents to provide care for their babies as much as possible.

We invite you to read the following information to find out if you are interested in participating in this study. You should know that your participation in this study is voluntary and that you can decide not to participate or change your decision and withdraw consent at any time, without altering the relationship with your medical team or causing any harm to your treatment.

**General information**

This is a project funded by the European Union through the “Horizon 2020” Framework Programme for Research and Innovation (Grant Agreement 101007922). The Madrilenial Health Service (SERMAS), through its La Paz University Hospital, acts as overall coordinator. Your hospital is part of the international set of partners covering neonatal units in Europe, Asia, Africa and North America.

**What is the purpose of the study?**

We want to know how the participation of parents in the care of their baby influences the child’s development as well as the way parents cope with the situation. For this, parents can participate in the care of their baby as soon as they are ready to do so, becoming part of the medical team from the first day. For this purpose, parents will receive adequate training to acquire all the necessary knowledge about their baby's environment and needs. This model of neonatal care is called Family Integrated Care (FICare).

**What is the background of the study?**

Research studies on the FICare model, the basis of the RISEinFAMILY project, have shown great benefits in newborns and their families, not only during hospital admission but also after discharge.

Newborn babies gained weight better, had a higher rate of breastfeeding, and a decrease in infections, less need for respiratory support, fewer days of hospitalisation and readmissions after discharge, and even better neurodevelopment in the first two years. Parents reported a decrease in stress and anxiety , as well as greater empowerment and self-confidence.

**How will the study be carried out?**

To evaluate the effect of the intervention we have to gather approximately a total of 1,500 babies and their families from the different hospitals. Part of these infant-family dyads will be trained in the FICare model, while others will provide data related to the health of newborns and their families before the implementation of the model, using the neonatal standard care. In this way we can compare standard neonatal care with FICare.

**What happens during the study?**

Every hospital that participates in this study will implement the FICare model in the Department of Neonatology. But not every hospital will start with it at the same time. The start time has been assigned taking into account the approximate time needed in each centre to undertake the appropriate internal reforms necessary for the implementation of the new care model. You may therefore be asked to participate in this study, even though the hospital where your child has been admitted has not yet started FICare. We would still like to know how you experience the hospital admission, and how you and your child are doing. In that case, your baby will be cared for through neonatal standard care.

If your hospital has already implemented FICare, you will receive a complete and individualised training for the care of your baby. This training will be carried out through a website where you will find all the necessary information, working with the nurse and your baby on a day-to-day basis and attending workshops on different topics that are important to you, either in person or online.

We will collect clinical data about your baby during admission and after discharge. The follow up will be continued up to 24 months corrected for prematurity.

In addition, we will ask you for information about the time spent on some of the tasks during admission. During your enrollment in this study, you will be invited to answer a series of online questionnaires to evaluate the psychological adaptation of the family and the possible changes to family life caused by the admission of the baby. Moreover, we would like to ask you for information about how having a baby admitted in our Department affects your economy in a general way (travel to the hospital, meals outside home etc.). These questionnaires will be given to you at the time of enrollment in the study, close to discharge and at 3-6 months of follow-up. Answering the questionnaires will of course be voluntary.

In most hospitals, there is a program for monitoring high-risk newborns after discharge. These follow-up visits will be used to collect the necessary data for the study about the evolution of your baby, thus avoiding additional visits. If the visits could not coincide, we would notify you in advance to try to obtain this information in a simple way.

**What risks does it have?**

In the studies carried out so far, no risks associated with the intervention of parents in the direct care of their children have been reported.

**If we participate, will we be informed of the results of the RISEinFAMILY project?**

Yes. If you decide that your child and you participate in the RISEinFAMILY project, we will keep in contact with you to communicate the results of the study when they are ready, if you wish.

The results of RISEinFAMILY, regardless of whether they are positive, negative or inconclusive, will be published and shared with parent organisations.

**What will happen to the data you have collected from my baby and me?**

The people involved in the study have no financial interest in relation to it. The sole purpose of the study is to improve the care of babies born prematurely or sick and their families. The investigators will have access to your baby's medical record for study purposes. Any information collected will be treated confidentially and safely guarded. Only duly authorised persons, participants in the study, will have access to your baby's data. Nevertheless, in order to allow study re-analysis, we will keep your data for at least 10 years. Finally, an anonymised dataset will be created and evaluated by data governing bodies. Anonymised data are not linkable to specific people and are crucial to allow further re-use in scientific works. If the anonymisation is evaluated as complete, the dataset will be offered to the scientific community in open access.

The use of personal data is governed by the data protection law of [country name] as detailed in a separate sheet [any partner should substitute the template for their own].

**Will you receive compensation if you participate in the study?**

Participation in the study will not cost you anything. However, your involvement in the intervention might represent changes in your daily agenda for expending more time than thought in the hospital during your baby’s admission. Neither will you get any compensation if you take part in this study.

**Ethics Committee**

The RISEinFAMILY project has an External Ethics Advisory Board to oversee the general conduct of the study. The study has been authorised by the Research Ethics Committee (CEim) of La Paz University Hospital and the corresponding [name of body] at [name of each hospital].

**How do I give consent for the study?**

You can first think carefully about this study. Then you tell the investigator if you understand the information and if you want to take part or not. If you want to take part, fill in the consent form that you can find with this information sheet. You and the investigator will both get a signed version of this consent form.

**Gratitude**

Thank you very much for the time you have taken to read this informational brochure. For any further information you require about RISEinFAMILY, please contact your doctor or nurse.

**Contact for RISEinFAMILY study**

Coordinator: Adelina Pellicer

Address: Department of Neonatology, La Paz University Hospital. Paseo de la Castellana 261, E-28046 Madrid, Spain.

Email: adelina.pellicer@salid.madrid.org

Telephone number: +34 91 727 74 16

Local contact person:

Address:

Email:

Telephone number:

**INFORMED CONSENT**

In relation to: “RISEinFAMILY_Integrating families at neonatal intensive care units for empowering them as primary caregivers.”

I have been asked to give consent for my child to participate in this medical study:

**Name of subject (child):**

− I have read the information sheet for parents/guardians. I was able to ask questions. My questions have been answered well enough. I had enough time to decide if I wanted my child to take part.

− I know that taking part is voluntary. I also know that I can decide at any time that my child and I will not take part after all. I do not have to explain why.

− I give consent to collect and use my child’s and my data. The investigators only do this to answer the question of this study.

**I have been informed by [investigator’s name]**

Signature: Date: __ / __ / __

I freely give my consent to participate in the study.

Parent’s/guardian’s name and SURNAME: as [relationship]

Email address

Signature: Date: __ / __ / __

Parent’s/guardian’s name and SURNAME: as [relationship]

Email address

Signature: Date: __ / __ / __

**CONFIDENTIALITY / DATA PROTECTION**

**CONSENT FOR RESEARCH STUDIES**

By means of this letter and in compliance with current regulations on data protection, I am informed of and expressly consent to the processing of the data from the medical records as well as those resulting from the participation in the study "RISEinFAMILY_Integrating families at neonatal intensive care units for empowering them as primary caregivers."

The Responsible for the Treatment is La Paz University Hospital (including Hospital Carlos III-Hospital Cantoblanco) whose Data Protection Delegate (DPD) is the “PDP Committee of the Department of Health of the Community of Madrid” with address at Paseo de la Castellana 280, 28046 Madrid (protecciondedatos.sanidad@madrid.org). The purpose is to establish the new family-centred neonatal care programs, called Family Integrated Care (FICare), which integrates parents in the direct care of their child.

The legal basis that legitimises the treatment is your consent, as well as the Statutory Act 3/2018, issued on December 5th, concerning Personal Data Protection and grant of Digital Rights; the Act 14/2007, of July 3, concerning Biomedical Research; and other current legislation on the matter. For this purpose, your data will be kept for the years necessary to comply with the obligations stipulated in the applicable current regulations, as well as while it is useful for the purpose for which it was obtained, and in any case, for at least five years.

Access to my personal information will be restricted to the study doctor(s), their collaborator(s) and other personnel who participate in it, health authorities, the Hospital's Research Ethics Committee and the promoter's monitors and auditors, who will be subject to the duty of secrecy inherent to their profession, when they need it, to check the data and procedures of the study, but always maintaining their confidentiality in accordance with current legislation. No additional data communications will be made, except in those cases required by law.

By providing your data you guarantee that you have read and expressly accepted their treatment as indicated. You may exercise your rights of access, rectification, deletion, opposition, limitation of treatment and portability, to the extent that they are applicable, through written communication to the Data Controller, with address at Hospital Universitario La Paz, Paseo de la Castellana 261, 28046 Madrid, specifying your request, along with your ID or equivalent document. Likewise, we inform you of the possibility of filing a claim with the Spanish Agency for Data Protection (C / Jorge Juan, 6 Madrid 28001 or www.agpd.es).

And for the record he signed it in Madrid at [month /day] 20___.

SIGNATURE:

Mr/Mrs ,

ID/Passport , in the name and representation of

**INFORMATION SHEET FOR HEALTHCARE PROFESSIONALS**

**Title of the study: Integrating families at neonatal intensive care units for empowering them as primary caregivers (RISEinFAMILY)**

**Introduction**

Dear colleagues,

We invite you to read the following information about RISEinFAMILY project to find out if you are interested in participating in this study. You should know that your participation in this study is voluntary and that you can decide not to participate or change your decision and withdraw consent at any time, without altering the relationship with your medical team.

**General information**

This is a project funded by the European Union through the “Horizon 2020” Framework Programme for Research and Innovation (Grant Agreement 101007922). The Madrilenial Health Service (SERMAS), through its La Paz University Hospital, acts as overall coordinator. Your hospital is part of the international set of partners covering neonatal units in Europe, Asia, Africa and North America.

**What is the purpose of the study?**

We want to know how the participation of parents in the care of their baby influences the child’s development as well as the way parents cope with the situation. For this, parents can participate in the care of their baby as soon as they are ready to do so, becoming part of the medical team from the first day. For this purpose, parents will receive adequate training to acquire all the necessary knowledge about their baby's environment and needs. This model of neonatal care is called Family Integrated Care (FICare).

In addition to exploring the viability and impact of the method on the health outcomes of high-risk newborns and on psychosocial aspects of families, we also want to know the role of the RISEinFAMILY model in self-care and satisfaction of the neonatal intensive care unit professional across multicultural environments with great socioeconomic diversity. We also want to explore the cost-effectiveness of this model of care through appropriate analyses.

**What is the background of the study?**

Research on the FICare model in health professionals is scarce and limited to small observational studies. Although preliminary data point to a decrease in professional burnout,it is important to further explore and gain more knowledge in this area.

**What happens during the study?**

To evaluate the effect of the intervention we have to gather approximately a total of 1,500 babies and their families from the different hospitals. During the first phase, prior to the implementation of the FICare model, the participants will provide data related to the health of the baby and the well-being of the families as a result of the current routine care (control group). Following this, at a later stage, infant-family dyads will be trained in the FICare model (intervention group). In this manner we will be able to compare the standard care currently in practice with the FICare model. Healthcare professionals will be invited to complete a specific questionnaire at two points: first, before the FICare model is implemented in their unit; second, at least 3 months after the full construction of the model in the corresponding unit. In some centres, where there is already experience in FICare, this questionnaire will only be passed once to each professional who participates in the study.

**How will the study be carried out?**

Every hospital that participates in this study will implement the FICare model in the Department of Neonatology. But not every hospital will start with it at the same time. The start time has been assigned taking into account the approximate time needed in each centre to undertake the appropriate internal reforms necessary for the implementation of the new care model. Therefore, some parents may be invited to participate in the study before the FICare model of care has been introduced at the hospital where their child has been admitted.

**What risks does it have?**

In the studies carried out so far, no risks associated with the intervention of parents in the direct care of their children have been reported.

If we participate, will we be informed of the results of the RISEinFAMILY project?

Yes. If you decide to participate in RISEinFAMILY project, we will keep in contact with you to communicate the results of the study when they are ready, if you wish.

The results of RISEinFAMILY, regardless of whether they are positive, negative or inconclusive, will be published and shared with parent organisations.

**What will happen to the data you have collected from my baby and me?**

The people involved in the study have no financial interest in relation to it. The sole purpose of the study is to improve the care of babies born prematurely or sick and their families, in addition to advancing the perceived quality of care offered by the healthcare professional. The investigators will have access to your medical record for study purposes. Any information collected will be treated confidentially and safely guarded. Only duly authorised persons, participants in the study, will have access to your data. Nevertheless, in order to allow study re-analysis, we will keep your data for at least 10 years. Finally, an anonymised dataset will be created and evaluated by data governing bodies. Anonymised data are not linkable to specific people and are crucial to allow further re-use in scientific works. If the anonymisation is evaluated as complete, the dataset will be offered to the scientific community in open access.

The use of personal data is governed by the data protection law of [country name] as detailed in a separate sheet [any partner should substitute the template for their own].

Will you receive compensation if you participate in the study?

Participation in the study will not cost you anything. Neither will you get any compensation if you take part in this study.

**Ethics Committee**

The RISEinFAMILY project has an External Ethics Advisory Board to oversee the general conduct of the study. The study has been authorised by the Research Ethics Committee (CEim) of La Paz University Hospital and the corresponding [name of body] at [name of each hospital].

**How do I give consent for the study?**

You can first think carefully about this study. Then you tell the investigator if you understand the information and if you want to take part or not. If you want to take part, fill in the consent form that you can find with this information sheet. You and the investigator will both get a signed version of this consent form.

**Gratitude**

Thank you very much for the time you have taken to read this informational brochure. For any further information you require about RISEinFAMILY, please contact your doctor or nurse.

**Contact for RISEinFAMILY study**

Coordinator: Adelina Pellicer

Address: Department of Neonatology, La Paz University Hospital. Paseo de la Castellana 261, E-28046 Madrid, Spain.

Email: adelina.pellicer@salud.madrid.org

Telephone number: +34 91 727 74 16

Local contact person:

Address:

Email:

Telephone number:

**INFORMED CONSENT**

In relation to: “RISEinFAMILY_Integrating families at neonatal intensive care units for empowering them as primary caregivers.”

I have been asked to give my consent to participate in this medical study:

**Name of subject:**

− I have read the information sheet for healthcare professionals. I was able to ask questions. My questions have been answered well enough. I had enough time to decide if I wanted to take part.

− I know that taking part is voluntary. I also know that I can decide at any time not take part after all. I do not have to explain why.

− I give consent to collect my data. The investigators only do this to answer the question of this study.

**I have been informed by [investigator’s name]**

Email address

Signature: Date: __ / __ / __

I freely give my consent to participate in the study.

NAME and SURNAME:

Email address

Signature: Date: __ / __ / __

**CONFIDENTIALITY / DATA PROTECTION**

**CONSENT FOR RESEARCH STUDIES**

By means of this letter and in compliance with current regulations on data protection, I am informed of and expressly consent to the processing of the data from the medical records as well as those resulting from the participation in the study "RISEinFAMILY_Integrating families at neonatal intensive care units for empowering them as primary caregivers."

The Responsible for the Treatment is La Paz University Hospital (including Hospital Carlos III-Hospital Cantoblanco) whose Data Protection Delegate (DPD) is the “PDP Committee of the Department of Health of the Community of Madrid” with address at Paseo de la Castellana 280, 28046 Madrid (protecciondedatos.sanidad@madrid.org). The purpose is to establish the new family-centred neonatal care programs, called Family Integrated Care (FICare), which integrates parents in the direct care of their child.

The legal basis that legitimises the treatment is your consent, as well as the Statutory Act 3/2018, issued on December 5th, concerning Personal Data Protection and grant of Digital Rights; the Act 14/2007, of July 3, concerning Biomedical Research; and other current legislation on the matter. For this purpose, your data will be kept for the years necessary to comply with the obligations stipulated in the applicable current regulations, as well as while it is useful for the purpose for which it was obtained, and in any case, for at least five years.

Access to my personal information will be restricted to the study doctor(s), their collaborator(s) and other personnel who participate in it, health authorities, the Hospital's Research Ethics Committee and the promoter's monitors and auditors, who will be subject to the duty of secrecy inherent to their profession, when they need it, to check the data and procedures of the study, but always maintaining their confidentiality in accordance with current legislation. No additional data communications will be made, except in those cases required by law.

By providing your data you guarantee that you have read and expressly accepted their treatment as indicated. You may exercise your rights of access, rectification, deletion, opposition, limitation of treatment and portability, to the extent that they are applicable, through written communication to the Data Controller, with address at Hospital Universitario La Paz, Paseo de la Castellana 261, 28046 Madrid, specifying your request, along with your ID or equivalent document. Likewise, we inform you of the possibility of filing a claim with the Spanish Agency for Data Protection (C / Jorge Juan, 6 Madrid 28001 or www.agpd.es).

And for the record he signed it in Madrid at [month /day] 20___.

SIGNATURE:

Mr/Mrs ,

ID/Passport , in the name and representation of
